# Efficacy of Single and Multi-Strain Probiotics on In Vitro Strain Compatibility, Pathogen Inhibition, Biofilm Formation Capability, and Stress Tolerance

**DOI:** 10.3390/biology11111644

**Published:** 2022-11-10

**Authors:** Puvaneswari Puvanasundram, Chou Min Chong, Suriana Sabri, Md Sabri Mohd Yusoff, Keng Chin Lim, Murni Karim

**Affiliations:** 1 Laboratory of Aquatic Animal Health and Therapeutics, Institute of Biosciences, University Putra Malaysia, Serdang 43400, Selangor, Malaysia; 2 Department of Aquaculture, Faculty of Agriculture, University Putra Malaysia, Serdang 43400, Selangor, Malaysia; 3 Enzyme and Technology Research Center, Faculty of Biotechnology and Biomolecular Sciences, University Putra Malaysia, Serdang 43400, Selangor, Malaysia; 4 Department of Veterinary Pathology and Microbiology, Faculty of Veterinary Medicine, Universiti Putra Malaysia, Serdang 43400, Selangor, Malaysia; 5 Laboratory of Sustainable Aquaculture, International Institute of Aquaculture and Aquatic Sciences, University Putra Malaysia, Port Dickson 71050, Negeri Sembilan, Malaysia

**Keywords:** multi-strain probiotics (MSP), single-strain probiotics (SSPs), antagonism, strain compatibility, properties, biofilm, stress tolerance

## Abstract

**Simple Summary:**

Probiotics are known to be a supplementary strategy which are one of the alternatives to prophylactic treatments such as chemical additives or antibiotics due to a higher demand for environmentally friendly tools to address disease outbreaks in aquaculture. The usage of multi-strain probiotic (MSP) has more benefits and increased potential on a host as opposed to the usage of a single-strain probiotic. The combination of few probiotic strains will help to maximize the benefit spectrum. In vitro screening steps to select a suitable probiotic strain as a candidate for an MSP is highly essential. The aim of this study is to screen and select probiotic strains that were isolated from various aquatic host to produce MSPs. From this study, two potential MSPs were selected based on criteria including the ability of the MSPs to inhibit common aquatic pathogens (*Streptococcus agalactiae*, *Aeromonas hydrophila*, *Vibrio harveyi*, *Vibrio parahaemolyticus*). These MSPs also showed beneficial characteristics that showed its potential to be applied on an aquatic host.

**Abstract:**

Compatibility of each strain in a multi-strain probiotic (MSP), along with its properties, becomes a strong base for its formulation. In this study, single-strain probiotics (SSPs) and multi-strain probiotics (MSPs) were evaluated in vitro for strain compatibility, microbial antagonism, biofilm formation capacity, and stress tolerance. *Bacillus amyloliquefaciens* L11, *Enterococcus hirae* LAB3, and *Lysinibacillus fusiformis* SPS11 were chosen as MSP1 candidates because they showed much stronger antagonism to *Aeromonas hydrophila* and *Streptococcus agalactiae* than a single probiotic. MSP 2 candidates were *Lysinibacillus fusiformis* strains SPS11, A1, and *Lysinibacillus sphaericus* strain NAS32 because the inhibition zone produced by MSP 2 against *Vibrio harveyi* and *Vibrio parahaemolyticus* was much higher than that produced by its constituent SSPs. MSP1 in the co-culture assay reduced (*p* < 0.05) *A. hydrophila* count from 9.89 ± 0.1 CFU mL^−1^ to 2.14 ± 0.2 CFU mL^−1^. The biofilm formation of both MSPs were significantly higher (*p* < 0.05) than its constituent SSPs and the pathogens. The SSPs in both MSPs generally showed resistance to high temperatures (80, 90, and 100 °C) and a wide range of pH (2 to 9). This in vitro assessment study demonstrates that MSP1 and 2 have the potential to be further explored as multi-strain probiotics on selected aquatic species.

## 1. Introduction

The use of probiotics for disease prevention and improved nutrition in aquaculture is becoming increasingly popular due to its beneficial properties that go beyond disease resistance. Over the years, various definitions of the term ‘probiotic’ have surfaced. Probiotics can be defined as live microorganisms which are either used singly or in combination to promote indigenous gut microbiota and the overall health of the host [1,2]. A more in-depth definition explores the detailed functions of probiotics, which include multiple improvements in disease resistance, growth performance, feed utilization, stress tolerance, and the well-being of the host via the improvement of intestinal microflora balance [3,4,5].

The gut is generally a complex environment and expecting maximum colonization from single-strain probiotics is highly impossible. Hence, the usage of MSPs has more benefits with increased potential on the host. In developing a mixture, it is necessary to know the specific or unique biological effects of each probiotic strain [6]. The mode of action of probiotics are species-specific due to the existence of interaction between aquatic animals and the microorganisms existing with animals as well as in their habitat [7]. The two main modes of action of probiotics in aquatic animals include competitive exclusion and immunomodulation [8].

Each probiotic strain has specific functionality that targets a particular disease. Hence, producing a mixed culture that consists of a combination of multi-strain or multi-species probiotics provides a niche where every probiotic strain complements each other’s benefit, leading to beneficial properties for the host [9]. This positive interrelationship between strains could be due to the exchange of different metabolites, which leads to higher diversity and a broader efficacy spectrum in an MSP [9,10].

The beneficial effects of probiotics are generally strain- or species-specific. Thus, the usage of MSPs could increase its effectiveness compared to using SSPs [9,11]. Hence, this study is aimed to screen SSPs through in vitro antagonistic assay. The SSPs used to produce MSP were chosen based on their ability to antagonize common aquatic pathogens (*Aeromonas hydrophila*, *Streptococcus agalactiae*, *Vibrio parahaemolyticus*, *Vibrio harveyi*). The MSPs used consist of three SSPs. The compatibility between strains is also taken into consideration when selecting a suitable SSP to produce MSP. These SSPs were previously isolated from different aquatic hosts. A few properties of the MSP, including biofilm formation and tolerance to a wide range of pH and temperature, were also determined in this study.

## 2. Materials and Methods

### 2.1. In Vitro Methods to Determine the Antimicrobial Effect of MSPs

#### 2.1.1. Broth Culture

All SSP isolates and pathogens were cultured on Tryptic Soy Agar (TSA) prior to the screening assay. The probiotic isolates used for screening to produce MSP were previously isolated from different aquatic hosts (Table 1). These isolates were provided by the Fish Health Laboratory, Department of Aquaculture, Faculty of Agriculture, Universiti Putra Malaysia (UPM). The pathogens used for the screening assay were *A. hydrophila, S. agalactiae, V. harveyi*, and *V. parahaemolyticus* (Table 2). *Streptococcus agalactiae* was provided by the Immunology Laboratory, Department of Aquaculture, Faculty of Agriculture, UPM. *Aeromonas hydrophila, V. harveyi*, *and V. parahaemolyticus* were provided by the Laboratory of Aquatic Animal Health and Therapeutics, Institute of Biosciences, UPM. A single colony from each probiotic isolates and pathogens was picked and transferred into Tryptic Soy Broth (TSB) individually. All broth cultures were incubated with agitation for 24 h at 30 °C.

#### 2.1.2. Antagonistic Assay—Well Diffusion Assay

The well diffusion assay was conducted to observe the antagonistic activity between probiotic isolates and pathogens [16]. Broth culture containing pathogen was diluted with ddH_2_O to 10^6^ CFU mL ^−1^. Then, the cotton bud was dipped into the diluted pathogen and swabbed evenly onto TSA until the surface was covered. Wells were then punched on the agar using a cork borer of 4 mm in diameter. Next, 10 µL of each SSPs (10^9^ CFU mL^−1^) was aliquoted into the well. The plates were then incubated for 16 h at 30 °C. After incubation, the inhibition zone was observed while measuring the diameter in mm.

The MSPs, which were used in the in vitro assay, consist of three SSPs each. The optical density of different probiotics cultured in the liquid medium was first measured at 550 nm to adjust the concentration before combining them to produce the MSPs. Multi-strain probiotics were then produced by adding equal volumes of three different SSPs. These MSPs were then mixed thoroughly and incubated in an incubator shaker for 30 min at 30 °C before being used for antagonism assay (well diffusion assay) against pathogens. The inhibition zone produced by the MSPs were observed and measured in mm.

#### 2.1.3. Compatibility Assessment by Well Diffusion Assay

Probiotic isolates used in the MSPs were screened for their compatibility with each other using a well diffusion assay. For compatibility assessment of two different probiotic isolates, a cotton bud was dipped into the broth culture containing the first probiotic (10^9^ CFU mL^−1^) and swabbed evenly onto TSA until the surface was covered. Wells were then punched on the agar using a cork borer of 4 mm in diameter. Next, 10 µL of the second probiotic (10^9^ CFU mL^−1^) were aliquoted into the wells. All plates were then incubated for 16 h at 30 °C. After incubation, the inhibition zone was observed and the diameter was measured in mm. Any observation of the inhibition zone signifies that the probiotic aliquoted into the wells inhibited the growth of the probiotic swabbed on the surface of TSA.

#### 2.1.4. Co-Culture Assay of MSP with Pathogens

The co-culture assay is a liquid medium assay used to observe and quantify the interaction between the potential MSP and pathogen over time [17]. The concentrations of potential MSP used were 10^4^, 10^5^, 10^6^, 10^7^, and 10^8^ CFU mL^−1^, whereas the concentration of pathogen used was 10^6^ CFU mL^−1^. Samples were taken at 0, 6, 12, 24, 48, and 96 h. At each sampling interval, 100 µL of the co-culture treatment was obtained and serially diluted for the ease of counting the colonies and proceeded to plate on selective media (Aeromonas Isolation Medium Base) (HiMedia, Mumbai) for *A. hydrophila* and Thiosulphate Citrate-Bile Salts-Sucrose agar (TCBS) (Difco Company, USA) for *V. harveyi* and *V. parahaemolyticus*. Suitable dilution (100 µL) was aliquoted onto the agar and spread using a sterile hockey stick. The spread plates were counted as colony forming unit per mL (CFU mL^−1^) using the following formula:

CFU mL^−1^ = (Number of colonies) × (Dilution factor)/[Volume of culture plate (mL)]

### 2.2. Properties of MSPs

#### 2.2.1. Biofilm Formation

The biofilm assay [18] was carried out to determine the ability of the MSP to produce biofilm. Non-sterile 96-well polyvinyl chloride culture plates were sterilized with 70% ethanol and air-dried in a sterile class II biosafety cabinet immediately prior to use. Tryptic soy broth (200 μL per well) was added and followed by the subsequent addition of 2 µL of SSP, MSP, and pathogen culture that was grown overnight at 30 °C (agitated at 200 rpm). Wells were covered and incubated without shaking for 18 h at 30 °C. Thereafter, 2 µL from each well (uniformly mixed) was transferred into triplicate wells containing 200 µL of fresh TSB and plates were incubated without shaking at 27 °C for 6, 12, 24, and 48 h, respectively. The supernatant was carefully discarded, and 150 µL of 1% crystal violet was aliquoted and incubated for 30 min at room temperature. The stain was removed, and the wells were washed twice with 175 µL sterile water. Crystal violet stain was solubilized by the addition of 175 µL of 95% ethanol to each well, and the absorbance was measured using a Fluorescence Spectra Viewer (Varioskan-Lux, Thermo Scientific Inc., Vantaa, Finland) adjusted to 595 nm (A_595_). The absorbance was adjusted by subtracting the A_595_ for wells that contained only media from the overall A_595_ for wells that contained the bacteria. The concentrations of crystal violet of SSPs, MSP, and pathogens were compared accordingly.

#### 2.2.2. Resistance to High Temperature

The ability of the bacteria isolates to resist different temperatures was assessed with slight modification [19]. Overnight culture of isolates was washed twice with PBS (pH 7.4) and then exposed to 80, 90, and 100 °C for 2, 5, and 10 min, respectively. An equal volume of sterile TSB was added to the heat-treated isolates to determine their ability to grow after the treatment. The bacterial culture was serially diluted before plating on the TSA for the ease of counting the colonies. Suitable dilution (100 µL) was aliquoted onto the TSA and spread using a sterile hockey stick. The bacterial growth was enumerated using the following formula:

CFU mL^−1^ = (Number of colonies) × (Dilution factor)/[Volume of culture plate (mL)]

#### 2.2.3. Tolerance of MSP towards Different pH

The optimal growth of MSP in various pH levels was assessed [20]. In brief, fresh overnight cultures of the MSP were inoculated into TSB broth with different pH values (2 to 9). The pH was adjusted with 2N HCl and 1N NaOH. The inoculated broths were incubated at 30 °C for 24 h, and growth was monitored using a UV spectrophotometer at 600 nm (OD) against the uninoculated broth.

### 2.3. Statistical Analysis

Statistical analysis was performed with IBM SPSS Statistic 20 software. All data collected were analyzed using one-way analysis of variance (ANOVA). Tukey’s test was applied for a pairwise comparison of the means. Data were expressed as mean ± standard error of the mean (SEM) at a significant level of *p* < 0.05.

## 3. Results

### 3.1. Antagonistic Assay

**Figure 1:**
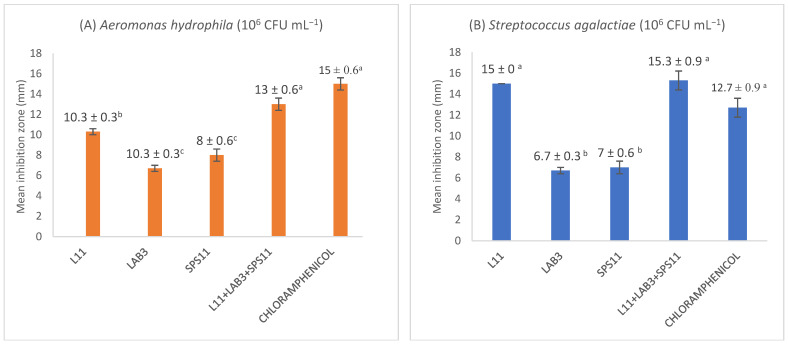

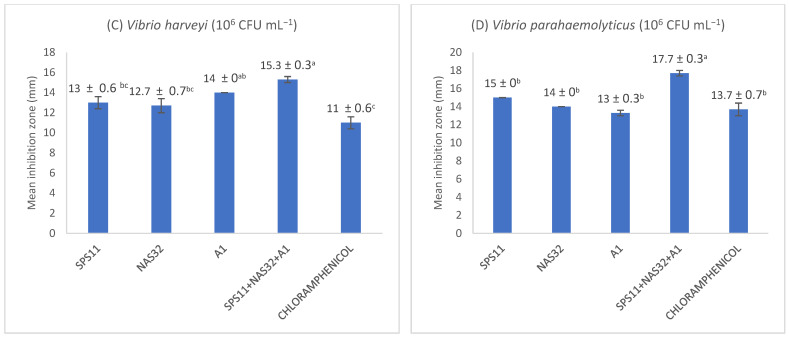
Agar well diffusion assay zone sizes for the inhibition of (**A**) *Aeromonas hydrophila* (10^6^ CFU mL^−1^), (**B**) *Streptococcus agalactiae* (10^6^ CFU mL^−1^), (**C**) *Vibrio harveyi* (10^6^ CFU mL^−1^), and (**D**) *Vibrio parahaemolyticus* (10^6^ CFU mL^−1^) using single- and multi-strain probiotics. L11 = *Bacillus amyloliquefaciens*; LAB3 = *Enterococcus hirae*; SPS11 = *Lysinibacillus fusiformis*; NAS32 = *Lysinibacillus sphaericus*; A1 = *Lysinibacillus fusiformis*; MSP1 = L11 + LAB3 + SPS11; MSP2 = SPS11 + NAS32 + A1; Chloramphenicol = Positive control. The values shown are the size of the inhibition zone ± SE (*n* = 3). Different alphabetical letters (^a, b, c, ab, bc^) indicate a statistically significant difference (*p* < 0.05).

All SSPs and MSPs (10^9^ CFU mL^−1^) were tested for their ability to inhibit the growth of *A. hydrophila*, *S. agalactiae*, *V. parahaemolyticus*, and *V. harveyi* at 10^6^ CFU mL^−1^. Results revealed that *B. amyloliquefaciens* (L11), *E. hirae* (LAB3), *L. fusiformis* (SPS11) inhibited the growth of *A. hydrophila* and *S. agalactiae* in the well diffusion assay. Hence, L11, LAB3, and SPS11 were mixed to produce MSP1 and screened in vitro against *A. hydrophila* and *S. agalactiae*. In the well diffusion assay, the inhibition zone (13 ± 0.6 mm) shown by MSP1 (L11 + LAB3 + SPS11) against *A. hydrophila* was significantly higher (*p* < 0.05) than its constituent SSPs (Figure 1A). The inhibitory zone of MSP1 (15.3 ± 0.9 mm) towards *S. agalactiae* was significantly higher (*p* < 0.05) than LAB3 and SPS11, but not L11 (Figure 1B).

SPS11, NAS32, and A1 exhibited antimicrobial activity against *V. harveyi*, and *V. parahaemolyticus*. SPS11, NAS32, and A1 showed more than 12 mm of inhibitory zones against both pathogens in well diffusion and spot assays. SPS11, NAS32, and A1 were mixed to produce MSP2 and screened against *V. parahaemolyticus*, and *V. harveyi*. Moreover, MSP2 produced a significantly higher (*p* < 0.05) inhibition zone towards *V. harveyi* (15.3 ± 0.3 mm) (Figure 1C) and *V. parahaemolyticus* (17.7 ± 0.3 mm) than its constituent SSPs (SPS11, NAS32, and A1) (Figure 1D).

### 3.2. Compatibility Assay between Strains

One criterion to consider when selecting probiotic isolates as candidates for MSP is the compatibility between strains. Through the compatibility assay, it was observed that the probiotic isolates L11, LAB3, and SPS11 were compatible with each other. Similarly, SPS11, NAS32, and A1 were also compatible with each other.

### 3.3. Co-Culture Assay

**Figure 2:**
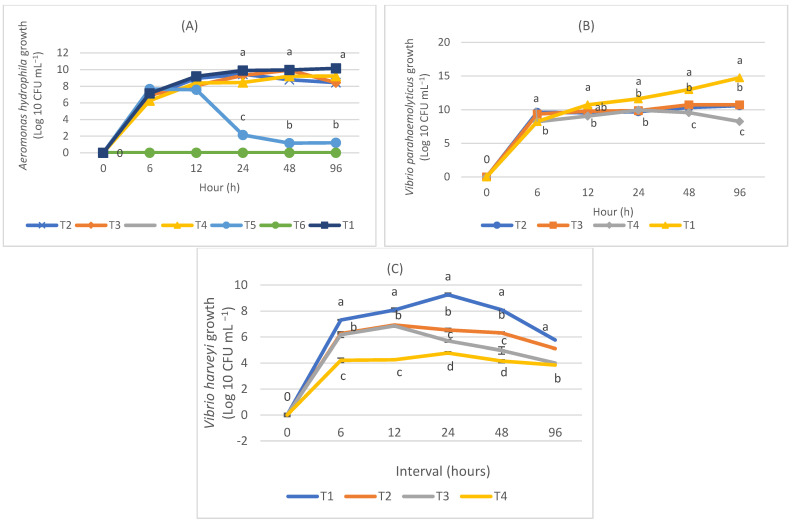
(**A**) The growth pattern of *A. hydrophila* incubated with different concentrations of MSP1 L11 + LAB3 + SPS11 (10^4^, 10^5^, 10^6^, 10^7^, and 10^8^ CFU mL^−1^) against time. T1 (*A. hydrophila* 10^6^ CFU mL^−1^), T2 (MSP 10^4^ CFU mL^−1^ + *A. hydrophila* 10^6^ CFU mL^−1^), T3 (MSP1 10^5^ CFU mL^−1^ + *A. hydrophila* 10^6^ CFU mL^−1^), T4 (MSP1 10^6^ CFU mL^−1^ + *A. hydrophila* 10^6^ CFU mL^−1^), T5 (MSP1 10^7^ CFU mL ^−1^+ *A. hydrophila* 10^6^ CFU mL^−1^), T6 (MSP1 10^8^ CFU mL^−1^ + *A. hydrophila* 10^6^ CFU mL^−1^); (**B**) The growth pattern of *V. parahaemolyticus* incubated with different concentrations of MSP2 SPS11 + NAS32 + A1 (10^4^, 10^6^, and 10^8^ CFU mL^−1^) against time. T1 (*V. parahaemolyticus* 10^6^ CFU mL^−1^), T2 (MSP2 10^4^ CFU mL^−1^ + *V. parahaemolyticus* 10^6^ CFU mL^−1^), T3 (MSP2 10^6^ CFU mL^−1^ + *V. parahaemolyticus* 10^6^ CFU mL^−1^), T4 (MSP2 10^8^ CFU mL^−1^ + *V. parahaemolyticus* 10^6^ CFU mL^−1^); (**C**) The growth pattern of *V. harveyi* incubated with different concentrations of MSP2 SPS11 + NAS32 + A1 (10^4^, 10^6^, and 10^8^ CFU mL^−1^) against time. T1 (*V. harveyi* 10^6^ CFU mL^−1^), T2 (MSP2 10^4^ CFU mL^−1^ + *V. harveyi* 10^6^ CFU mL^−1^), T3 (MSP2 10^6^ CFU mL^−1^ + *V. harveyi* 10^6^ CFU mL^−1^), T4 (MSP2 10^8^ CFU mL^−1^ + *V. harveyi* 10^6^ CFU mL^−1^). Different alphabets (^a, b, c, ab^) indicate significant differences among treatments (*p* < 0.05).

The number of *A. hydrophila* after co-cultured with MSP1 at the concentration of 10^7^ CFU mL^−1^ was significantly reduced (*p* < 0.05) at 24-, 48-, and 96-h intervals (Figure 2A). The growth of *A. hydrophila* at 24-h was 2.14 ± 0.2^c^ CFU mL^−1^, which showed a significant reduction (*p* < 0.05) from the control treatment (9.89 ± 0.1 CFU^a^ mL^−1^). Similarly, at 48 and 96 h, MSP1 at 10^7^ CFU mL^−1^ significantly reduced *Aeromonas* count from 9.96 ± 0.1^a^ CFU mL^−1^ and 10.15 ± 0.3^a^ CFU mL^−1^ to 1.15 ± 0.7^b^ CFU mL^−1^ and 1.20 ± 0.8^b^ CFUmL^−1^, respectively. There was no growth of *A. hydrophila* observed in the treatment with MSP1 at concentration 10^8^ CFU mL^−1^, signifying total inhibition of pathogen by the MSP.

The number of *V. parahaemolyticus* after co-cultured with MSP2 at concentration 10^8^ CFU mL^−1^ showed a significant reduction (*p* < 0.05), starting from the 6-h interval (Figure 2B). MSP2 at all concentrations (10^4^, 10^6^, and 10^8^ CFU mL^−1^) showed a significant reduction (*p* < 0.05) in *Vibrio* count at the 24-h interval. The most significant reduction of *V. parahaemolyticus* load by MSP2 was from 14.74 ± 0.2 CFU mL^−1^ to 8.23 ± 0.9 CFU mL^−1^ at a 96-h interval. On the other hand, MSP2 co-cultured with *V. harveyi* (10^6^ CFU mL^−1^) significantly reduced (*p* < 0.05) pathogen count at all intervals (Figure 2C). The highest reduction in *V. harveyi* count was at a 24-h interval from 9.26 ± 0.15 CFU mL^¬1^ to 4.77 ± 0 CFU mL^−1^.

### 3.4. Biofilm Formation

**Figure 3:**
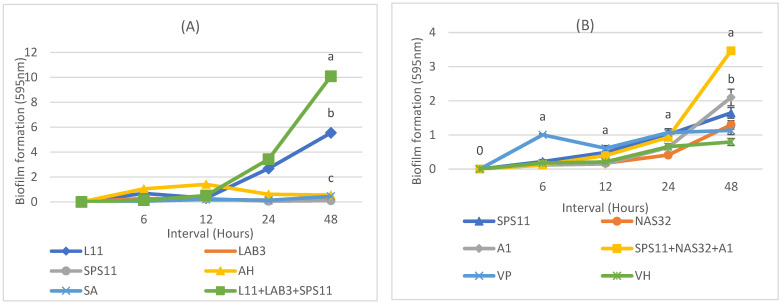
(**A**) Comparison of biofilm formation for SSPs (L11, LAB3, and SPS11) and MSP1 (L11 + LAB3 + SPS11) with *A. hydrophila* and *S. agalactiae*. (**B**) Comparison of biofilm formation for SSP (SPS11, NAS32, and A1), MSP (SPS11 + NAS32 + A1) with pathogen *V. parahaemolyticus* and *V. harveyi*. Biofilm formation was measured at intervals of 6, 12, 24, and 48 h. Different alphabets indicate significant differences among treatments (*p* < 0.05).

The biofilm production by MSP1 (L11 + LAB3 + SPS11) was compared with its constituent SSPs (L11, LAB3, SPS11) and pathogens (*A. hydrophila* and *S. agalactiae*). Results showed that the biofilm production by MSP1 was significantly higher (*p* < 0.05) than its constituent SSPs and pathogens (Figure 3A). At a 24-h interval, MSP1 produced significantly more biofilm (3.42 ± 0.2 nm) (*p* < 0.05) than L11 (2.68 ± 0.17 nm), LAB3 (0.151 ± 0.04 nm), and SPS11 (0.0594 ± 0.01 nm) (Table 3). MSP1 produced the highest biofilm at a 48-h interval (10.09 ± 0.4 nm). Both *A. hydrophila* and *S. agalactiae* recorded low biofilm formation at 24- and 48-h intervals.

The biofilm formation by MSP 2 (SPS11 + NAS32 + A1) was also compared with its constituent SSPs (SPS11, NAS32, A1) and pathogens (*V. parahaemolyticus* and *V. harveyi*). Results showed that the biofilm production by MSP2 was significantly higher (*p* < 0.05) than its constituent SSPs and pathogens (Figure 3B). At a 48-h interval, MSP 2 produced significantly more biofilm (3.46 ± 0.01 nm) (*p* < 0.05) than SPS11 (1.65 ± 0.16 nm), NAS32 (1.29 ± 0.13 nm), and A1 (2.10 ± 0.24 nm) (Table 4).

### 3.5. Resistance of Single-Strain Probiotics (SSPs) to High Temperatures

**Figure 4:**
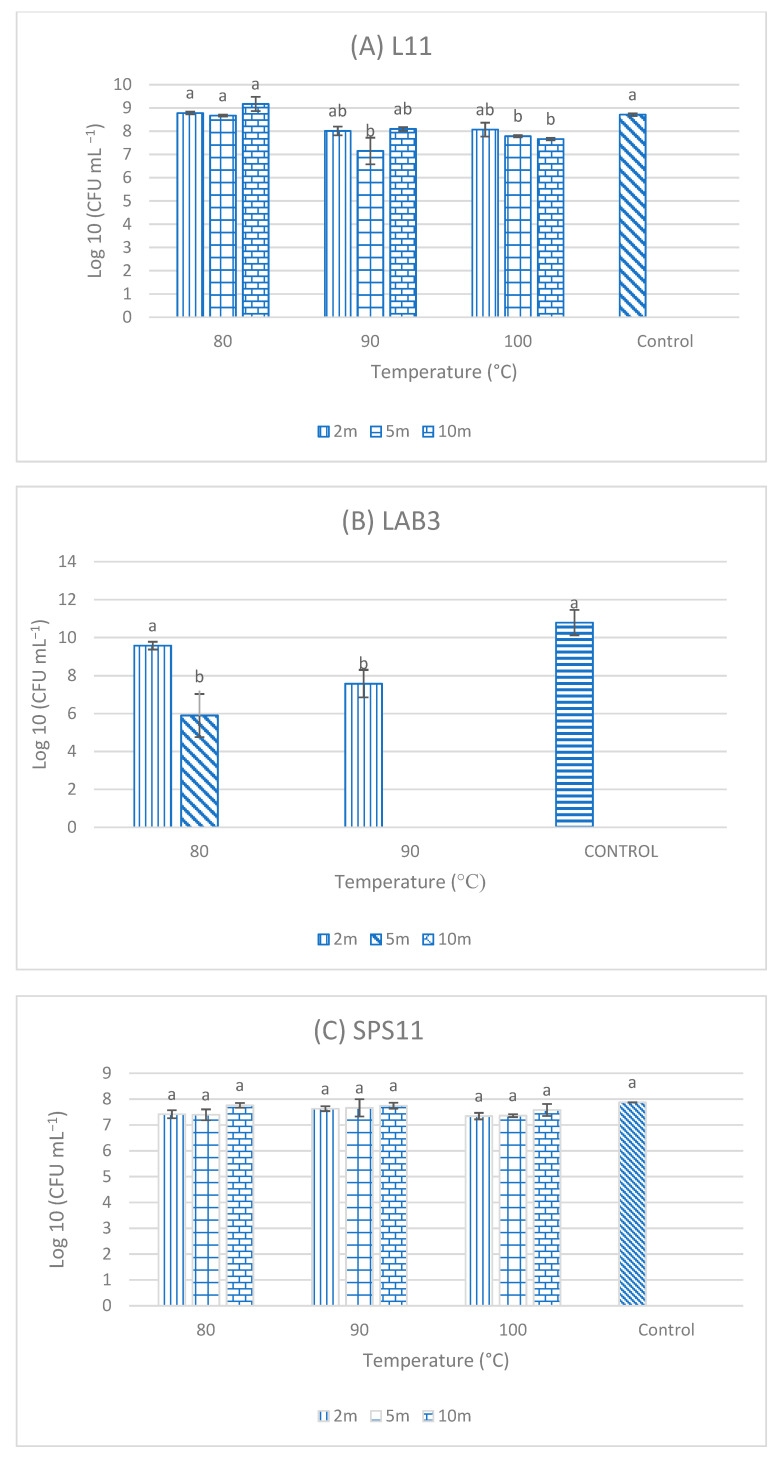

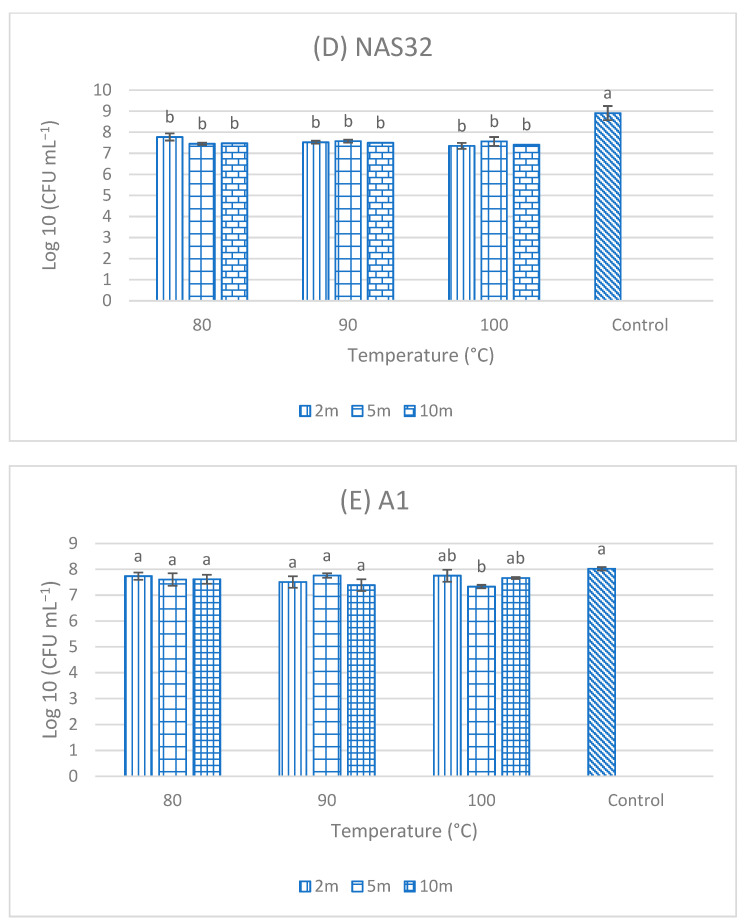
Resistance of (**A**) L11, *Bacillus amyloliquefaciens*; (**B**) LAB3, *Enterococcus hirae*; (**C**) SPS11, *Lysinibacillus fusiformis*; (**D**) NAS32, *Lysinibacillus sphaericus*; and (**E**) A1, *Lysinibacillus fusiformis* to high temperatures. Values are presented as mean ± SE (*n* = 3). Significant differences are indicated by different letters (*p* < 0.05).

After exposure to different temperatures (80, 90, and 100 °C) for 2, 5, and 10 min, respectively, the three SSP isolates (L11, LAB3, and SPS11), which were the candidate for MSP1, showed the best results. High growth was observed in L11 isolate after exposure to high temperatures (Figure 4) and it was not significantly different (*p* > 0.05) from the control (isolates without exposure to higher temperatures), except for exposure to 90 °C for 5 min and to 100 °C for 5- and 10-min. High growth was also observed in SPS11 exposed to the various temperatures in comparison to the control. However, no significant differences (*p* >0.05) were observed among different exposure times to high temperatures. As for LAB3, high growth was observed when exposed to 80 °C for 2 and 5 min as well as those exposed to 90 °C for 2 min. No growth was observed when LAB3 isolated were exposed to 100 °C. NAS32 and A1 were able to withstand high temperature as there was growth observed in all three temperatures across all time intervals.

### 3.6. Tolerance of MSP towards Different pH Values

**Figure 5:**
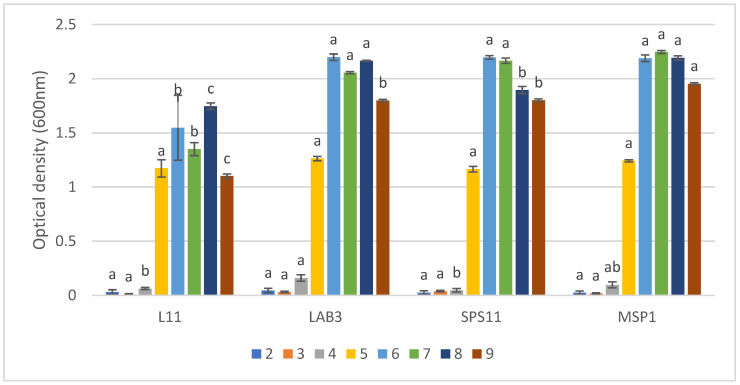
The growth of MSP1 and its constituent single-strain probiotics (SSPs) at different pH levels (2–9). Values are presented as mean ± SE (*n* = 3). Significant differences are indicated by different letters (*p* < 0.05).

Results showed that the growth (optical density) of MSP1 changed significantly (*p* < 0.05) from pH 2 to 9. This MSP showed slightly slow growth at pH 2, increased until pH 7, then decreased to pH 9 (Figure 5). The growth of SSPs and MSP1 was not significantly different at pH 2, 3, 4, and 5. The growth of LAB3, SPS11, and MSP1 in pH 6, 7, and 8 was significantly higher than L11. MSP1 showed the highest significant growth in pH 9.

**Figure 6:**
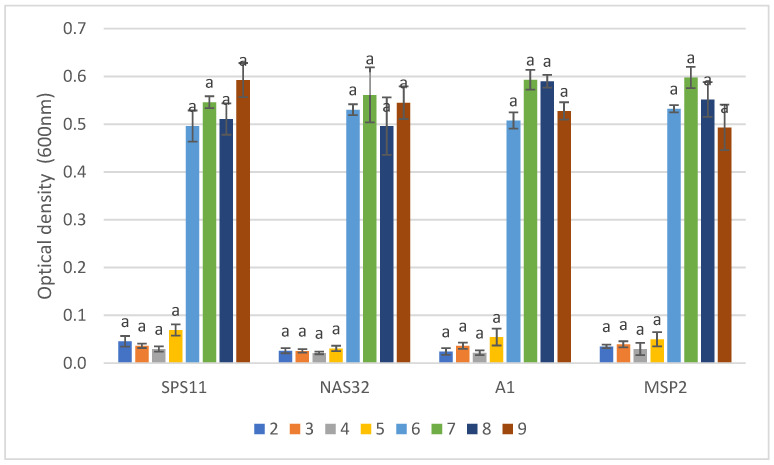
The growth of MSP2 and its constituent single-strain probiotics (SSPs) at different pH levels (2–9). Values are presented as mean ± SE (*n* = 3). Significant differences are indicated by different letters (*p* < 0.05).

There were no significant differences observed in the growth of MSP2 compared to its constituent SSPs across pH 2 to 9 (Figure 6). The highest growth of MSP 2 was observed at pH 7 and the lowest growth was observed at pH 4. The optimum growth of SPS11 was in pH 9 whereas, for NAS32 and A1, it was in pH 7.

## 4. Discussion

Multi-strain probiotics (MSP) have been used in aquaculture to improve aquatic animal growth, illness resistance, and immune response while also enhancing water quality variables [21,22,23,24,25]. To construct a functional MSP, a few prerequisites must be met. The usage of probiotics from mucosal layer of aquatic animals is preferred over those isolated from terrestrial sources [26]. This is because the efficacy of a gut microbiota is dependent on a microorganism’s ability to interact within the gastrointestinal system, thereby helping the host by influencing its biological functions. [26,27]. As a result, the utilization of probiotic isolates from aquatic origin, such as the one employed in this study, is highly promising.

It has been demonstrated that supplementing a host with a mixture of various probiotics in combination with other bacteria improves fish tolerance to pathogens [6,21,28,29,30]. The act of combining beneficial bacteria would result in an increase inhibition activity towards pathogen adhesion [28,31]. MSP1 and 2 strains come from a variety of genera, including *Bacillus*, *Lysinibacillus*, and *Enterococcus*. Although the use of probiotics from the genus *Enterococcus* is uncommon in aquaculture, earlier research has examined this species and demonstrated its potential as a probiotic in aquaculture [32,33,34]. For instance, the use of *E. hirae* enhanced the juvenile hybrid catfish in terms of growth and survival when challenged with *A. hydrophila* [32].

The effect of one probiotic appeared to inhibit more than one pathogen. MSP1 in this study, which consists of L11, LAB3, and SPS11, showed antimicrobial activity against *S. agalactiae* and *A. hydrophila*. Likewise, MSP2 (SPS11, NAS32, and A1) exhibited antagonism against the growth of both *V. harveyi* and *V. parahaemolyticus*. Comparably, *Bacillus* sp. and *Aeromonas sobriaa* were effective in reducing the mortality of rainbow trout challenged with different types of pathogens (*Aeromonas salmonicida*, *Lactococcus garvieae*, *Streptococcus iniae*, *Vibrio angullarum*, *Vibrio ordalii*, and *Yersinia ruckerii*) [35]. A much more recent work by [36] deduced that *Enterococcus* sp. isolated from the intestine of parrot fish (*Sparisome viride*) inhibited both *A. hydrophila* and *V. harveyi*, which was tested using in vitro well diffusion assay.

Similar to this study, *Bacillus amyloliquefaciens* isolated from the digestive tract of *Oreochromis niloticus* showed antimicrobial activity against both *A. hydrophila* and *S. agalactiae* [37]. Furthermore, *B. amyloliquefaciens* isolated from the intestine of Indian major carp (*Catla catla*) and supplemented at a concentration of 1 × 10^9^ CFU g^−1^ exerted a significant effect on relative percent survival (RPS) of rohu fish compared to control treatment, which showed disease symptoms of *Aeromonas* [38]. Previously, this probiotic species has also been used as a candidate for MSP. The incorporation of *B. amyloliquefaciens* and *B. pumilus* mixture into feed (5 × 10^8^ CFU mL^−1^) protected *Pangasianodon hypothalamus* against *Edwardsiella ictaluri* infection [39]. It could be considered that multi-species probiotic can have a greater inhibitory effect on pathogen than single strains that included in that mixture.

In this study, L11 (*B. amyloliquefaciens*), LAB3 (*E. hirae*), and SPS11 (*L. fusiformis*) showed antagonism against the growth of *A. hydrophila* and *S. agalactiae*, which are two common freshwater pathogens. There is evidence that marine probiotics such as the one utilized in this study can reduce mortality caused by *A. hydrophila. Lactobacillus plantarum*, a marine lactic acid bacteria, showed high resistance to *A. hydrophila* infection in Nile tilapia with higher RPS than the control group. Moreover, fish fed with the probiotic showed no clinical signs of disease [40]. *Lysinibacillus sphaericus* strain PKA17 isolated from the gut of Asian catfish showed antagonism against *Vibrio vulnificus* and *V. parahaemolyticus* [41]. A mixed probiotic consisting of *L. fusiformis* and *Bacillus megaterium* showed antagonism activity against *Vibrio alginolyticus* in both in vitro and preliminary in vivo assay using *Artemia* culture [42].

Based on the results of a competitive assay using co-culture method, MSP1 and MSP2 successfully inhibited the growth of the respective pathogens significantly. The antimicrobial property of the isolates in both MSPs could be due to antagonistic compounds that are toxic (bactericidal) or inhibitory (bacteriostatic) towards other microorganisms [43].

The first aim of this study was to assess the compatibility of probiotic strains, which will provide the foundation for creating an MSP. This is based on the hypothesis that a bacterium (the producer) creates an extracellular chemical that inhibits itself or another bacterial strain (the indicator). In this study, strain L11 had no inhibitory activity against *E. hirae* (LAB3) or *L. fusiformis* (SPS11). SPS11, NAS32, and A1 probiotics isolated from microalgae, on the other hand, were not inhibitory to each other. Even though mutual inhibition of strains is one of the most important aspects to consider when developing a probiotic mix, it does not limit the MSP’s effectiveness [44].

The results showed that MSP 1 was able to develop more biofilm than pathogens in *in vitro*, which was one of the characteristics of the MSP examined (*A. hydrophila* and *S. agalactiae*). Similarly, MSP2 produced more biofilm than pathogens (*V. parahaemolyticus* and *V. harveyi*). Hence, the results indicated that the MSP 1 produced by combining SSPs (L11, LAB3, and SPS11) and MSP2 produced by SPS11, NAS32, and A1 harbor a synergistic effect to deliver a higher amount of biofilm, which competes with the pathogen for adhesion sites.

The mechanism of biofilm production by probiotic strains is to outcompete pathogenic bacteria for nutrients and habitat colonization. This suggests that when MSP1 and MSP2 are supplemented to a host, it may provide functionality in competing against the pathogen in the gut, thus conferring protection to the host. Biofilms are multicellular complexes surrounded by an extracellular matrix bound together and formed in the aqueous system, which acts as a layer with a boundary to a solid phase [45]. The bacteria immobilized in a biofilm are known to be in a stationary phase and are assumed to produce bioactive compounds that protect the bacteria from outgrowth by other bacteria. There are many successful attempts at the beneficial use of biofilms [46]. Isolates that have been found to have antipathogenic properties could also be able to colonize the fish digestive tract, such as *B. amyloliquefaciens* isolated from *Labeo calbasu*, which showed antagonism towards *A. hydrophila* and showed positive outcomes in biofilm formation assay [20]. This is similar to the findings in this study, where both MSP1 and 2 inhibited a wide range of pathogens and produced biofilm when tested *in vitro*. In recent years, other methods of microbial antagonistic mechanisms that do not directly kill pathogens have been investigated. In a study that compares the biofilm formation of a few probiotic strains and pathogens, the probiotic strains of *Pseudoalteromonas* sp., *Reugeria* sp., and *Bacteroides* sp. were better biofilm formers as some of these strains formed biofilms as early as 12 h compared to the pathogen (*Vibrio* sp.), which produced a maximum biofilm density after 48 h [47].

One significant property that distinguishes a bacterial strain as a probiotic is its ability to enhance colonization resistance (CR) in the host intestine against prospective infections. Because the survivability of probiotics in the host is questionable due to various extreme conditions such as acidic stomach, incorporating multiple strains in a mixed probiotic could help in enhancing the probiotics’ chances of at least partial survival [9]. In theory, the MSP creates a probiotic niche, which could aid in the colonization of the strain harmed by severe circumstances [9].

For a probiotic to be utilized as a feed additive, it must be able to withstand high temperatures since the feed production process requires high temperatures [19]. In this study, all SSP isolates from MSP1 and MSP 2 were able to resist high temperatures up to a certain extent. Similarly, *Bacillus velezensis*, *Bacillus subtilis*, and *B. amyloliquefaciens* isolated from Nile tilapia had higher viability after heat treatment (80, 90, and 100 °C) [37]. The high viability of the strains after heat treatment in this study strongly suggests that they could be used as feed additives. Heat treatment is an essential process during feed production to eliminate pathogenic cells [19]. The application of thermotolerant probiotics that can withstand stress during the processing and storage of products in aquaculture is of great interest [48]. High temperature resistance test performed on *B. velezensis* showed high survival rate of the strain after exposure to 80 °C for 2, 5, and 10 min [49]. Likewise, *Bacillus methylotrophicus* LSG2-3-2 and *Bacillus tequilensis* LSG3-6 could also tolerate high temperatures up to 80 °C [50].

Tolerance to a wide pH range is one of the characteristics that aid in probiotic colonization and survival in the host’s gut. [37]. Potential probiotics should be able to survive in both low and high pH conditions [51]. The common stomach pH value in *O. niloticus* is 0.9 to 7.0 [52]. In the present study, all strains in MSP 1 and 2 were able to grow at pH 2 to 7. Similarly, *Lysinibacillus* sp. isolated from Nile tilapia can survive at lower pH values (1 and 3) [53]. During pepsin activity in the intestinal activity of fish, pH changes ranging from 1 to 7.8 occurs, and pH values higher than 7.8 occur during lipid activity [54]. In our study, the highest growth observed for strain L11 was at pH 8, whereas for strain LAB3 and SPS11 were at pH 6. On the other hand, the highest growth observed for strain SPS11 was at pH 9 and for strain NAS32 and A1 were at pH 7. Similar to the findings in this study, *Lysinibacillus* species isolated from gastrointestinal tract of Nile tilapia is tolerant to pH value of 1, 3, and 7 [53]. Lactic acid bacteria, *Enterococcus avium*, isolated from tiger shrimp was able to survive in acidic to basic pH (1.5–7.2) [55]. Gradual increase of *B. amyloliquefaciens* isolate within pH range from 1 to 7 followed by decrease in growth from pH 7 to 10, which showed the ability of isolates to survive in extreme acidic and alkaline condition [20]. More than 50% of isolate *B. velezensis* survived after the exposure to pH 2, 3, 4, and 5 [49].

The main idea of using MSPs is that more diseases can be targeted by using a mixture of strains [10]. A mixed culture that contains a multiple-strain of probiotics may complement each other’s health effects, which leads to synergistic properties [9].

## 5. Conclusions

Probiotics are beneficial microorganisms that exert multiple benefits in every aspect of aquaculture through various modes of action. The concept of MSP is to maximize the effectiveness of probiotic action by applying more than a single strain of probiotic to the host. Therefore, the formulation of MSP requires careful consideration of every aspect, including the compatibility of strains and the properties of MSP, to obtain the maximum yield of MSP. Although in terms of antagonism of pathogen, out of all 3 SSPs, L11 has shown the highest inhibitory effect against pathogen, the combination of all 3 strains did portray a broader efficacy in the aspect of beneficial properties portrayed through biofilm formation capability. This is because more strains give more chances of success for a beneficial effect on hosts. Hence, multi-strain probiotics 1 (L11 + LAB3 + SPS11) showed a positive inhibitory effect against the growth of *A. hydrophila* and *S. agalactiae* in vitro. Likewise, MSP2 (SPS11 + NAS32 + A1) showed inhibitory activity against the growth of *V. parahaemolyticus* and *V. harveyi*. These MSPs have also exhibited properties, which include the ability to form high biofilm and withstand a wide range of pH and high temperature. These characteristics make the MSPs a good candidate for application as probiotics in aquaculture. Although in vitro testing of this MSP has shown promising results, any in vitro testing of probiotics should be followed by an in vivo testing on specific host species. Hence, based on the property of these MSPs, it can be incorporated into feed before being supplemented to the host.

## Figures and Tables

**Table 1:** Single-strain probiotics (SSPs) isolated from various aquatic hosts.

No	Isolate	Name	Genbank Accession Number	Host	Reference
1	L11	*Bacillus* *amyloliquefaciens*	MN096657	*Portunus pelagicus* (Blue swimmer crab)	[12]
2	LAB3	*Enterococcus* *hirae*	MK757970	*Lates calcarifer* (Asian seabass)	[13]
3	SPS11	*Lysinibacillus* *fusiformis*	MK757974	*Spirulina sp*. (Microalgae)	[14]
4	NAS32	*Lysinibacillus sphaericus*	MK757973	*Nannochloropsis sp*. (Microalgae)	[14]
5	A1	*Lysinibacillus* *fusiformis*	MK764897	*Amphora* sp. (Microalgae)	[15]

**Table 2:** List of pathogens used in the screening assay.

No	Isolates	Host	Provided by
1	*Aeromonas hydrophila*	*Oreochromis* sp.	Laboratory of Aquatic Animal Health and Therapeutics, Institute of Biosciences, UPM
2	*Streptococcus agalactiae* type 3	*Oreochromis* sp.	Immunology Laboratory, Department of Aquaculture, Faculty of Agriculture, UPM
3	*Vibrio parahaemolyticus* strain VPK1	*Epinephelus fuscoguttus*	Laboratory of Aquatic Animal Health and Therapeutics, Institute of Biosciences, UPM
4	*Vibrio harveyi* strain VH1	*Epinephelus fuscoguttus*	

**Table 3:** Biofilm-forming ability of SSPs, pathogens and MSP1 at different time interval.

	Interval			
Isolates	6	12	24	48
L11	0.688 ± 0.22 ^ab^	0.303 ± 0.08 ^b^	2.68 ± 0.17 ^b^	5.54 ± 0.37 ^b^
LAB3	0.273 ± 0.18 ^bc^	0.170 ± 0.02 ^b^	0.151 ± 0.04 ^cd^	0.151 ± 0.01 ^c^
SPS11	0.15 ± 0.00 ^c^	0.264 ± 0.04 ^b^	0.0594 ± 0.01 ^d^	0.098 ± 0.02 ^c^
*A. hydrophila*	1.05 ± 0.03 ^d^	1.41 ± 0.03 ^c^	0.615 ± 0.03 ^c^	0.551 ± 0.15 ^c^
*S. agalactiae*	0.052 ± 0.01 ^c^	0.198 ± 0.04 ^b^	0.124 ±0.03 ^d^	0.445 ± 0.03 ^c^
MSP1	0.138 ±0.01 ^c^	0.509 ± 0.16 ^b^	3.42 ± 0.17 ^a^	10.09 ± 0.4 ^a^

Different alphabetical letters (^a, b, c, d, ab, bc, cd^) indicate a statistically significant difference (*p* < 0.05).

**Table 4:** Biofilm-forming ability of SSPs, pathogens and MSP2 at different time interval.

	Interval			
Isolates	6	12	24	48
SPS11	0.219 ± 0.06 ^b^	0.485 ± 0.03 ^ab^	1.01 ± 0.13 ^ab^	1.65 ± 0.16 ^bc^
NAS32	0.128 ± 0.02 ^b^	0.172 ± 0.00 ^d^	0.413 ± 0.03 ^c^	1.29 ± 0.13 ^cd^
A1	0.118 ± 0.01 ^b^	0.15 ± 0.00 ^d^	0.633 ± 0.05 ^bc^	2.10 ± 0.24 ^b^
*V. parahaemolyticus*	1.01 ± 0.03 ^a^	0.609 ±0.08 ^a^	1.07 ± 0.12 ^a^	1.13 ± 0.12 ^cd^
*V. harveyi*	0.177 ± 0.01 ^b^	0.208 ± 0.03 ^cd^	0.655 ± 0.09 ^bc^	0.793 ± 0.09 ^d^
MSP2	0.123 ± 0.02 ^b^	0.396 ± 0.06 ^bc^	0.927 ± 0.03 ^a^	3.46 ± 0.01 ^a^

Different alphabetical letters (^a, b, c, d, ab, bc, cd^) indicate a statistically significant difference (*p* < 0.05), Different superscript indicates significant differences (*p* < 0.05).

## Data Availability

Not applicable.
